# Non-linear parameters of specific resistance loops to characterise obstructive airways diseases

**DOI:** 10.1186/s12931-016-0484-7

**Published:** 2017-01-09

**Authors:** Marko Topalovic, Vasileios Exadaktylos, Thierry Troosters, Geert Celis, Jean-Marie Aerts, Wim Janssens

**Affiliations:** 1 Laboratory of Respiratory Diseases, University Hospital Leuven, Department of Clinical and Experimental Medicine, KU Leuven, Herestraat 49, 3000 Leuven, Belgium; 2 Division Animal and Human Health Engineering, Department of Biosystems, Faculty of Bioscience Engineering, KU Leuven, Leuven, Belgium; 3 Department of Rehabilitation Sciences, Faculty of Kinesiology and Rehabilitation, KU Leuven, Leuven, Belgium

**Keywords:** Body-plethysmography, Airway resistance, Pulmonary function tests, Chronic obstructive pulmonary disease, Asthma

## Abstract

**Background:**

Specific resistance loops appear in different shapes influenced by different resistive properties of the airways, yet their descriptive ability is compressed to a single parameter - its slope. We aimed to develop new parameters reflecting the various shapes of the loop and to explore their potential in the characterisation of obstructive airways diseases.

**Methods:**

Our study included 134 subjects: Healthy controls (*N* = 22), Asthma with non-obstructive lung function (*N* = 22) and COPD of all disease stages (*N* = 90). Different shapes were described by geometrical and second-order transfer function parameters.

**Results:**

Our parameters demonstrated no difference between asthma and healthy controls groups, but were significantly different (*p* < 0.0001) from the patients with COPD. Grouping mild COPD subjects by an open or not-open shape of the resistance loop revealed significant differences of loop parameters and classical lung function parameters. Multiple logistic regression indicated RV/TLC as the only predictor of loop opening with OR = 1.157, 95% CI (1.064–1.267), *p*-value = 0.0006 and R^2^ = 0.35. Inducing airway narrowing in asthma gave equal shape measures as in COPD non-openers, but with a decreased slope (*p* < 0.0001).

**Conclusion:**

This study introduces new parameters calculated from the resistance loops which may correlate with different phenotypes of obstructive airways diseases.

## Background

Obstructive lung diseases, predominantly asthma and COPD, are a group of respiratory diseases characterized by airflow limitation [1, 2]. The primary pathophysiologic impairment in these diseases is an increase of airways resistance that originates from mucosal and submucosal inflammation, bronchial constriction and airway collapse during expiration. The rise in airways resistance requires greater intrapleural pressure changes to provide sufficient pressure gradients for the initiation and maintenance of airflow. Airway resistance particularly increases during expiration when positive intrathoracic pressures are further compressing the intraluminal space of the airways [3].

**Fig. 1:**
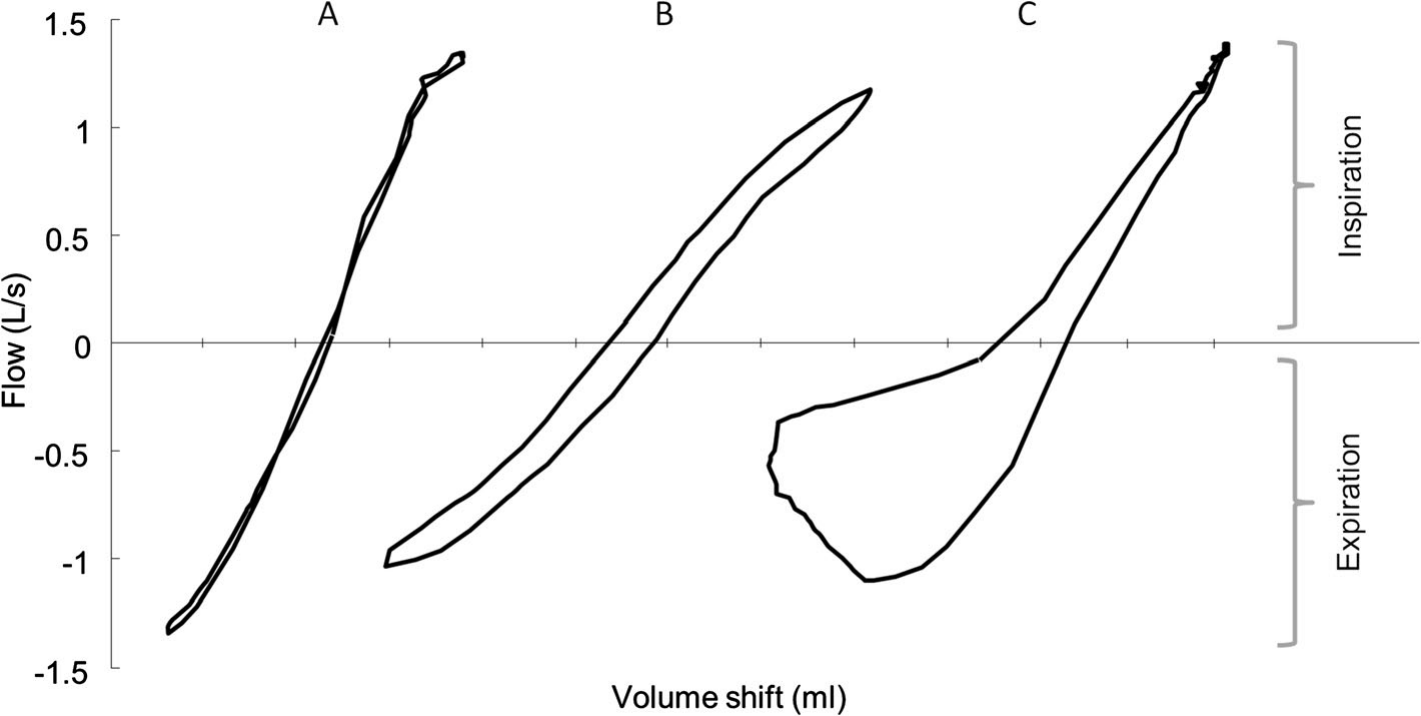
Comparison of typical specific resistance loops: **a** Healthy subject, **b** Asthma subject, **c** COPD subject; Examples of asthma and COPD are having almost identical sG_AW_ (=0.60 [1/ kPa*sec]), yet they are visually completely different due to different resistive mechanisms

From a diagnostic point of view spirometry is considered as the gold standard to diagnose COPD and asthma, as well as to assess the level of airway obstruction [4, 5]. Spirometry requires forced maximal manoeuvres but cannot quantify increased resistance of the airways at tidal breathing, which may be characteristic and specific for the underlying disease. Whole-body plethysmography, however, allows the computation of airways resistance by measuring alveolar pressure changes and corresponding airway flows during tidal breathing in a closed body box [6]. More specifically, plethysmography records the small changes in box pressure by compression and decompression of thoracic gas which correspond to small volume changes, also known as shift volume. Simultaneously, it records airflow at the mouth generated by these subtle pressure changes. The relationship between these two measurements is linearly expressed as specific airways conductance (sG_AW_) or specific airways resistance (sR_AW_) [7]. Hence, the diagnostic power of resistance loops is reduced to one single parameter, the slope of the linear relation, rather than to its differences in shape which are affected by central or peripheral airway obstruction, expiratory collapse or end-expiratory airway closing, and which may vary in function of the breathing manoeuver and the thoracic gas volume (Fig. 1) [6, 7]. As demonstrated in our previous work, the slope of these loops is lacking discriminatory ability. It only marginally contributes to the diagnosis and differentiation of COPD and asthma [8].

Different studies have identified some limitations of the current parameterization of specific resistance loops, mainly by its variability, the necessity for artefact corrections and the clear evidence that the slope is significantly determined by small changes in the breathing frequency [9–11]. Similar observations were done with the forced oscillation technique (FOT), demonstrating important within and between-breath variation for which should be corrected to identify real airway patency or obstruction [12–15]. Other researchers have described methodologies to optimize the slope estimation as a key parameter for the calculation of airways resistance with plethysmography [7, 16, 17]. However, to the best of our knowledge, no one has explored the potential of other parameters that may better reflect the curvilinear two-dimensional shape of plethysmographic resistance loops.

We hypothesize that a more detailed mathematical modelling of the tidal breathing loops may yield new parameters which better represent the non-linear dynamics of specific resistance curves. With an empirical model-based approach our first objective was to develop geometrical parameters reflecting the shape of the loop. Secondly, we designed second-order mathematical models linking shift volumes to airflows during breathing. Finally, we checked whether these newly developed parameters associated with other important lung function measures in different clinical phenotypes of obstructive airways diseases.

## Methods

### Study subjects

For development of different mathematical models, lung function data of 134 subjects were retrospectively collected from a databank of the outpatient clinic of the University Hospital of Leuven (Belgium) based on an established diagnosis of asthma (*N* = 22) or COPD (*N* = 90) and compared with a healthy control group (*N* = 22). All enrolled subjects were Caucasians between 19 and 84 years old who had performed complete pulmonary function testing (including post-bronchodilator spirometry, whole-body plethysmography for lung volumes and airway resistance, and diffusing capacity). All patients provided informed consent for the use of lung function and clinical data and the protocol was approved by the ethics committee of the University Hospital of Leuven. Baseline characteristics of the subjects are presented in the Table 1: i) Healthy group is defined as a group of asymptomatic subjects without smoking history with all lung function parameters falling in the normal reference range; ii) Asthma group: subjects with or without smoking history, a previous clinical diagnosis of asthma based on symptoms and therapy response, with a non-obstructive lung function (post bronchodilator FEV_1_/FVC ratio > 0.7) and positive reaction on inhaled methacholine (FEV_1_ drop of at least 20% at maximal concentration of 8 mg/ml of methacholine) [18]; and iii) COPD group: subjects with at least 10 pack-years of smoking history and post-bronchodilator FEV_1_/FVC ratio below 0.7 [19]. In a subgroup of asthma subjects (*N* = 11), a methacholine challenge test was repeated with pre and post resistance and lung volume measurements.

**Table 1:** Study population characteristics

	Healthy	Asthma	COPD
Subjects, n	22	22	90
Gender, M/F	16/6	13/9	67/23
Age, years	60 (59–63)	34 (22–49)	65 (58–71)
BMI, kg/m^2^	24.8 (22.9–27.1)	23.9 (22.3–26.9)	23.6 (21.0–27.8)
FEV_1_, %predicted	114 (105–124)	107 (93–123)	47 (30–72)
FEV_1_/FVC, %	74.5 (71.8–77.5)	77 (73–82)	46.5 (32–58)
DL,_CO_, %predicted	94 (81–100)	88 (81–99)	51 (39–67)
RV, %predicted	101 (93–113)	104 (92–112)	147 (121–190)
TLC, %predicted	112 (106–118)	106 (96–113)	109 (99–127)
FRC, %predicted	120 (102–128)	112 (103–120)	142 (122–184)
RV/TLC, %	34.4 (32.2–36.8)	27.4 (24.0–32.9)	56.3 (41.6–62.7)
R_AW_, %predicted	86 (76–107)	94 (77–109)	239 (122–333)
sG_AW_, %predicted	155 (131–192)	128 (108–171)	47 (27–98)

Values are median and IQR

Definition of abbreviations: *BMI* body mass index, *DL*_*,CO*_ carbon monoxide diffusing capacity, *FEV*_*1*_ forced expiratory volume in one second, *FRC* functional residual capacity, *RV* residual volume, *R*_*AW*_ airway resistance, *sG*_*AW*_ specific airway conductance, *TLC* total lung capacity

### Pulmonary function tests

All pulmonary function tests were performed according to the American Thoracic Society (ATS)/ European Respiratory Society (ERS) criteria [20] using standardized equipment (Masterscreen Jaeger, Carefusion, Germany). Spirometry data are post-bronchodilator measures and are expressed, along with pre-bronchodilator plethysmography measurements of airway resistance and lung volume as percent predicted of normal reference values [21, 22]. Diffusing capacity (DL,_CO_) was measured by the single-breath carbon monoxide gas transfer method and expressed as percent predicted of reference values [23].

### Geometrical modelling

To geometrically explain the opening of the resistance loops we developed a series of new parameters that may capture what is visually apparent to the observer. From each expiratory resistance loop, using the same electronic data and MATLAB software as in the transfer function development, the following shape descriptive parameters were derived:

1. **Area of the expiratory loop:** it stands for total surface covered by the expiratory phase of the breathing manoeuver (Fig. 2, panel I.A). Area of loop (AOL) *per se* is effort depended, as deeper breathing will cause larger loop surfaces. Therefore, we normalized each loop to a range [0, 1] for both flows and shift volumes.

**Fig. 2:**
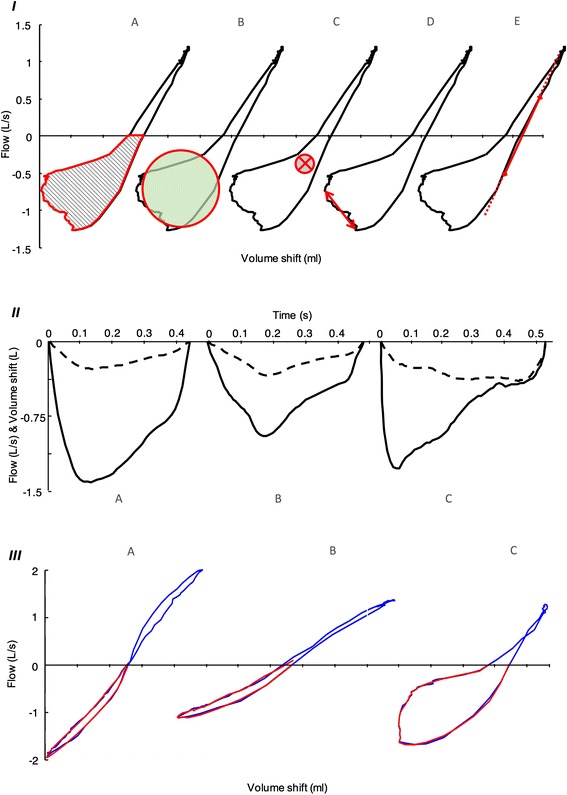
**Panel I** Geometrical parameters in COPD subject: *a* Area of the loop, *b* Roundness, *c* Median point, *d* Asynchrony, *e* sG_0.5_. **Panel II** Examples of input–output relationship presented over time: *a* Healthy subject, *b* Asthma subject, *c* COPD subject; Solid line represents flow (model output), dashed line is volume shift (model input). Transfer function model explains how input transforms to output. **Panel III** Visualisation of model performance with modelled expiration (*red line*) over the original loop (*blue line*): *a* Healthy subject (NMRSE = 95%), *b* Post methacholine asthma subject (NMRSE = 92%), *c* COPD subject (NMRSE = 94%)
2. **Roundness of the expiratory loop:** In essence, this parameter measures how closely the expiratory loop approaches to a circle (Fig. 2, panel I.B). It appears in an interval from 0 to 1, where 0 means complete closure of the loop (flat line) while 1 means a perfect circle. Smooth eclipses will have a low roundness and an open loop is never expected to reach a perfect circle. Roundness (Rnd) is defined based on (Eq. 1):
  1 $$ Rnd=\frac{4\uppi \ast \mathrm{A}\mathrm{O}\mathrm{L}}{\mathrm{Perimete}{\mathrm{r}}^2} $$
  where AOL represents the complete area of the normalised expiratory loop, and Perimeter is the perimeter of the same loop.
3. **Median point of the expiratory loop:** it is a two dimensional measure, with the values coming from both, X axis (median volume shift = point X) and Y axis (median flow = point Y) in the expiratory phase (Fig. 2, panel I.C). Ordinarily, both axes have a negative value as somewhat longer expiration and larger opening of the loop pulls the point lower to the third quadrant.
4. **Asynchrony between volume shift and flow:** The nonlinearity in the relationship between volume shift and flow lies in the asynchrony between this two factors. It corresponds to airflow drops despite increasing volume shifts (Fig. 2, panel I.D, it can be also depicted from Fig. 2, panel II.C). Asynchrony is a measure expressed as time difference of volume shift peak and peak of the flow.
5. **SG**_**0.5**_ **:** Similar to sG_AW_, yet limited to the linear drop between right-handside inspiratory and expiratory flow rates of ±0.5 L/s (Fig. 2, panel I.E.) [6].

### Transfer function modelling

Development of the data-based input–output transfer function models was performed in an offline framework in MATLAB (8.3, The MathWorks, Natick, Massachusetts) using the System Identification Toolbox [24]. From the Masterlab system, measurements of tidal expiration were exported at a sampling rate of 100Hz. Shape differences of specific resistance loop were described via relationship of their two creating factors: flow and volume shift. Volume shift was used as a model input, while the result of that generated pressure, flow, was used as a model output (input–output relationship shown in Fig. 2, panel II).

To characterize the breathing process and explain the data in a parametrically efficient way and yet sustain simplicity in the sense of model parameters and model order, an iterative system identification procedure was used [25]. Based on this procedure we have chosen a second order discrete-time transfer function (TF) model (Eq. 2) as most the appropriate.

2 $$ H(z)=\frac{F(z)}{Vs(z)}=\frac{b_0+{b}_1{z}^{-1}+{b}_2{z}^{-2}}{1+{a}_1{z}^{-1}+{a}_2{z}^{-2}} $$

where F stands for Flow and Vs for Volume shift. Coefficients *a*_*1*_, *a*_*2*_ and coefficients *b*_*0*_, *b*_*1*_ and *b*_*2*_ are the TF denominator and numerator, respectively. Z is a discrete domain operator. From the defined second order model, we further extracted process descriptive parameters:

1. the steady state gain (*SSG*), defined as a ratio of the steady state output and the input (Eq. 3), which in simplified sense represents volume shift multiplication to reach a certain flow level in steady state conditions.
  3 $$ SSG=\frac{\varDelta F(z)}{\varDelta Vs(z)}=\frac{{\displaystyle {\sum}_{i=0}^2{b}_i}}{1+{\displaystyle {\sum}_{i=1}^2{a}_i}} $$
2. using denominator coefficients, two dynamic components of tidal expiration were derived: namely *Pole*_*1*_ and *Pole*_*2*_ (i.e. the roots of the denominator) (Eq. 4).
  4 $$ Pol{e}_{1,2} = \frac{a_1\pm \sqrt{{a_1}^2-4{a}_2}}{2} $$
3. finally, the numerator coefficients (*b*_*0*_ *, b*_*1*_ *, and b*_*2*_), were used for group comparison.

### Statistical analysis

JMP Pro version 12, (SAS Institute, Cary, USA) was used to perform statistical analysis. The Shapiro-Wilk test was used to inspect normality of the groups. To control differences between two groups (paired or unpaired, where appropriate) with parametric and non-parametric distribution T-test and Mann–Whitney test were used, respectively. In the case of multiple group comparisons, ANOVA or Kruskal-Wallis tests were performed, depending of group distribution. The logistic-regression model was applied for binary variables analyses, where in addition stepwise selection was used to identify the subset of variables that had the strongest relation to outcome, using default criteria of significance at the 0.25 level to enter and 0.5 level to leave the model. The model consisted of lung function parameters: FVC, %pred, FEV_1_, %pred, FEV_1_/FVC, %pred, PEF, %pred. TLC, %pred, RV, %pred, FRC, %pred., DL,_CO_, %pred, K_CO_, %pred., RV/TLC. To determine normal value range, two-sided prediction interval of 99% was applied.

## Results

### Models

The assessment of the geometrical parameters was possible with the data of all subjects. Due to instable results of TF model parameters (|z| > 1), one healthy and ten COPD subjects were excluded from further analysis [26]. In general, confirmation of the appropriate model selection was demonstrated with a high goodness of fit expressed as normalized root mean square error (NRMSE) of 90 (85–92)% (values are median and IQR) for complete dataset. A visualisation of the model performance with its accuracy is shown in Fig. 2, Panel III.

Table 2 depicts the median values of all resistive parameters in each group, revealing significant differences in the majority of parameters when comparing COPD with Asthma or Healthy controls, and no significant differences between non-obstructive asthmatics and healthy controls. As expected, all geometrical parameters reflecting the opening of the loop (Roundness, Area of loop and Asynchrony) are significantly different in COPD. Point X and *b’s* from TF confirm that with airways obstruction in COPD, an increased pressure (increased volume shift) has to be generated to maintain tidal breathing without significant changes in flow (Point Y). This is coherent with the measuring standard of whole-body plethysmography where the subjects are instructed for a tidal breathing at 1 Hz at FRC securing similar flows for everyone (disease independent).

**Table 2:** Resistive parameters in different groups

	Healthy	Asthma	COPD	*p* value (H vs. A)	*p* value (H vs. C)	*p* value (A vs. C)
Roundness	0.04 (0.03–0.07)	0.07 (0.04–0.09)	0.41 (0.17–0.58)	0.7900	<0.0001	<0.0001
Area of Loop	0.07 (0.05–0.10)	0.12 (0.08–0.16)	0.38 (0.21–0.51)	0.9189	<0.0001	<0.0001
Asynchrony, msec	10 (0–20)	15 (0–33)	70 (30–140)	0.9064	<0.0001	<0.0001
Point X, ml	−29 (−67–−15)	−26 (−79–−6)	−128 (−318–−48)	0.9999	0.0006	0.0005
Point Y, ml/sec	−243 (−470–−25)	−195 (−440–37)	−331 (−443–−209)	0.9653	0.5014	0.5014
*b*_*0*_	5.66 (3.78–7.43)	5.77 (4.64–7.09)	1.51 (0.84–3.27)	0.9999	<0.0001	<0.0001
*b*_*1*_	−8.88 (−11.8–−5.0)	−8.57 (−11.5–−5.6)	−2.36 (−4.43–−0.17)	0.8356	<0.0001	<0.0001
*b*_*2*_	4.22 (2.39–5.21)	3.19 (1.10–4.97)	0.90 (−0.18–1.82)	0.9999	<0.0001	<0.0001
*SSG*, 1/sec	5.48 (4.62–6.21)	5.18 (4.11–6.16)	1.34 (0.62–2.61)	0.9999	<0.0001	<0.0001
*Pole1*	0.94 (0.90–0.97)	0.95 (0.89–0.97)	0.91 (0.82–0.96)	0.9999	0.4899	0.4068
*Pole2*	0.93 (0.80–0.96)	0.92 (0.59–0.95)	0.78 (0.53–0.91)	0.6674	0.0241	0.7444
sG_AW_, 1/ kPa*sec	1.32 (1.12–1.63)	1.09 (0.92–1.45)	0.40 (0.23–0.83)	0.9635	<0.0001	<0.0001
sG_0.5_ , 1/ kPa*sec	1.42 (1.15–1.57)	1.33 (1.24–1.47)	0.75 (0.49–1.14)	0.9999	<0.0001	<0.0001

Values are median and IQR

Definition of abbreviations: *Point X* Median point based on median volume shift values, *Point Y* Median point based on median flow values

### Resistance loop in COPD

As shown in Table 2, most resistance loops of COPD opened to an imperfect circular shape with significant differences on roundness, normalized AOL and asynchrony. As multivariate analysis indicated that roundness from all resistive parameters was the best differentiator between non-obstructive subjects (asthma and healthy) and COPD, roundness was chosen as the key variable to describe opening of the loop (data not shown). When applying a 99% confidence interval to define the normal range of roundness in healthy and non-obstructive asthma subjects, a 0.18 cut-off came out as the upper limit of normality. When using this > 0.18 cut-off for the definition of loop opening in COPD, 67/90 patients were considered as “Openers” whereas 23 were defined as “Non-openers”. When comparing common lung function parameters between the group of “Openers” and “Non-openers” in COPD, it was found that 22 of 23 “Non-openers” were present in the mild to moderate stages (I and II) of COPD and that those subjects were characterized by a significantly higher FEV_1_, a lower RV and RV/TLC ratio compared to the “openers” of GOLD stage I and II (Table 3). Multiple logistic regression with the stepwise selection demonstrated that in COPD GOLD I and II, RV/TLC ratio was the only predictor for loop opening with an OR of 1.157 (95% CI of (1.064–1.267), *p* value of 0.0006 and model R^2^ = 0.35). When looking at all COPD stages, again RV/TLC associated best with loop opening with an OR of 1.168 (95% CI of (1.092–1.259), *p* value <0.0001 and model R^2^ = 0.61) and a small contribution of FEV_1_/FVC ratio (OR (CL) 0.866 (0.746–0.949), *p* value 0.0004, adding to total R^2^ = 0.72). Data presented in Table 4.

**Table 3:** Comparison of patients with opened vs. unopened loop in mild to moderate COPD

	Non-openers	Openers	*p* value
Subjects, n	22	17	
BMI, kg/m^2^	24.9 (22.0–29.4)	26.8 (24.1–27.8)	0.6093
FEV_1_, %predicted	83 (76–93)	65 (61–76)	0.0018
DL_CO_, %predicted	67 (55–79)	69 (53–75)	0.6774
RV, %predicted	115 (106–133)	134 (120–150)	0.0368
TLC, %predicted	109 (99–115)	107 (102–110)	0.6793
FRC, %predicted	122 (101–135)	131 (120–142)	0.1578
RV/TLC, %	38.7 (35.7–40.4)	46.6 (41.5–53.9)	0.0007
sG_AW_, 1/ kPa*sec	1.03 (0.90–1.21)	0.61 (0.46–0.74)	<0.0001
sG_0.5_ , 1/ kPa*sec	1.23 (1.06–1.38)	1.00 (0.81–1.15)	0.0047
Roundness	0.09 (0.06–0.12)	0.31 (0.22–0.41)	<0.0001
Area of Loop	0.14 (0.10–0.20)	0.31 (0.23–0.44)	<0.0001
Asynchrony, msec	10 (0–40)	40 (30–70)	0.0097
Point X, ml	−38 (−93–−19)	−92 (−209–−31)	0.0827
Point Y, ml/sec	−251 (−379–−177)	−251 (−406–−139)	0.7107
*b*_*0*_	4.10 (2.92–5.56)	1.63 (1.18–2.58)	0.0013
*b*_*1*_	−3.47 (−8.11–1.16)	−2.40 (−3.95–0.20)	0.3080
*b*_*2*_	1.42 (−2.87–3.27)	0.77 (−0.46–1.97)	0.5132
*SSG*, 1/sec	4.03 (2.76–4.54)	1.67 (1.50–2.21)	<0.0001
*Pole1*	0.84 (0.67–0.96)	0.93 (0.82–0.97)	0.2301
*Pole2*	0.64 (0.26–0.89)	0.86 (0.49–0.95)	0.1216

Values are median and IQR; Differences between groups controlled with T-test or Mann–Whitney test depending of distribution

**Table 4:** Relationship between pulmonary function parameters and the opening of resistance loops in multiple logistic regression with stepwise selection: A/ in mild to moderate COPD, B/ in all COPD

Variables	Odds Ratio (95% Confidence limit)	*p* value
Mild COPD		
RV/TLC, %	1.157 (1.064–1.267)	0.0006
COPD		
RV/TLC, %	1.168 (1.092–1.259)	<0.0001
FEV_1_/FVC, %	0.866 (0.746–0.949)	0.0004

In table are shown only significant variables, not excluded by a stepwise selection. The model consisted of lung function parameters: FVC, %pred, FEV_1_, %pred, FEV_1_/FVC, %pred, PEF, %pred. TLC, %pred, RV, %pred, FRC, %pred., DL,_CO_, %pred, K_CO_, %pred., RV/TLC

### Changes in resistance loops during bronchoconstriction

In a subgroup of non-obstructive asthma patients (*N* = 11), airway narrowing was induced by increasing levels of methacholine during a challenge test. In all patients FEV_1_ dropped below 20% of its initial value at methacholine concentrations <4 mg/ml, indicative for acute bronchoconstriction with subsequent rises in airways resistance. These changes were confirmed by a significant decrease of specific conductance (sG_AW_ and sG_0.5_), a significant increase in median volume shift (point X) and *b*_*0*_ and significant drop in *b*_*1*_ and *SSG* (data shown in Table 5). Although the median roundness statistically increased in the post-methacholine challenge conditions (from 0.08 to 0.13), these small changes were considered not to be relevant as in 7/11 patients roundness stayed under the upper limit of “Non-openers”. Additionally, normalized AOL and asynchrony did not change, indicating that differences in the slope and changes in input related variables (point X, *b*_*0*_, *b*_*1*_, *SSG*) were perfectly in phase with changes in output (flow).

**Table 5:** Lung function and resistance parameters in the subgroup of asthma patients with pre and post methacholine challenge test

	Pre	Post	*p* value
Subjects, n	11	11	
Openers, n	0	4	
FEV_1_, %predicted	108 (91–115)	75 (67–89)	<0.0001
RV, %predicted	107 (102–118)	132 (115–162)	0.0188
TLC, %predicted	106 (95–113)	102 (89–115)	0.2014
FRC, %predicted	116 (110–120)	130 (123–140)	0.0508
RV/TLC, %	27.4 (26.4–32.5)	32.1 (27.9–48.4)	0.1289
sG_AW_, 1/ kPa*sec	1.02 (0.88–1.10)	0.45 (0.38–0.51)	0.0010
sG_0.5_ , 1/ kPa*sec	1.28 (1.24–1.34)	0.63 (0.57–0.69)	0.0010
Roundness	0.08 (0.02–0.09)	0.13 (0.12–0.27)	0.0029
Area of Loop	0.14 (0.02–0.18)	0.14 (0.13–0.19)	0.1678
Asynchrony, msec	20 (0–20)	20 (20–50)	0.1339
Point X, ml	−14 (−27–1)	−115 (−172–3)	0.0137
Point Y, ml/sec	−158 (−188–73)	−258 (−399–−42)	0.2447
*b*_*0*_	6.04 (4.68–7.03)	2.03 (1.67–4.11)	0.0049
*b*_*1*_	−7.72 (−11.14–−5.20)	−2.37 (−4.45–0.06)	0.0105
*b*_*2*_	2.92 (0.87–4.15)	0.91 (−0.88–2.09)	0.1242
*SSG*, 1/sec	5.00 (4.16–5.89)	2.13 (1.41–2.90)	0.0020
*Pole1*	0.95 (0.86–0.97)	0.90 (0.57–0.94)	0.3203
*Pole2*	0.85 (0.16–0.95)	0.71 (0.45–0.91)	0.8984

Values are median and IQR; Differences between groups controlled with T-test or Mann–Whitney test depending of distribution

Finally, when comparing resistive and lung function parameters of obstructed asthma (post methacholine) with mild to moderate COPD “Openers” and “Non-openers”, it was clear that obstructive asthma patients presented with the lowest slopes and highest point X for only little increases of roundness, asynchrony and AOL. By contrast, COPD openers presented with the highest roundness, asynchrony and AOL. Consistent with previous findings, not FEV_1_ (*p* = 0.093) but RV/TLC ratio (*p* < 0.0001) was the main clinical lung function determinant that differentiated between obstructive asthma and COPD openers (see Table 6).

**Table 6:** Comparison of groups where flow limitation is undoubted

	Post methacholine Asthma (A)	Non-Openers mild COPD (B)	Openers mild COPD (C)	*p* value (A vs. B)	*p* value (A vs. C)
Subjects, n	11	22	17		
FEV_1_, %predicted	75 (67–89)	83 (76–93)	65 (61–76)	0.3757	0.0937
FEV_1_/FVC, %	65 (60–69)	62 (58–68)	55 (48–61)	0.7242	0.0027
RV/TLC, %	32.1 (27.9–48.4)	38.7 (35.7–40.4)	46.6 (41.5–53.9)	<0.0001	<0.0001
sG_AW_, 1/ kPa*sec	0.45 (0.38–0.51)	1.03 (0.90–1.21)	0.61 (0.46–0.74)	<0.0001	0.2420
sG_0.5_ , 1/ kPa*sec	0.63 (0.57–0.69)	1.23 (1.06–1.38)	1.00 (0.81–1.15)	<0.0001	0.0033
Roundness	0.13 (0.12–0.27)	0.09 (0.06–0.12)	0.31 (0.22–0.41)	0.0309	0.0656
Area of Loop	0.14 (0.13–0.19)	0.14 (0.10–0.20)	0.31 (0.23–0.44)	0.6732	<0.0001
*SSG*, 1/sec	2.13 (1.41–2.90)	4.03 (2.76–4.54)	1.67 (1.50–2.21)	0.0022	0.2651
Point X, ml	−115 (−172–3)	−38 (−93–−19)	−92 (−29–−31)	-	-
Asynchrony, msec	20 (20–50)	10 (0–43)	40 (30–65)	0.9999	0.4266

Values are median and IQR; Differences between groups controlled with ANOVA or Kruskal-Wallis test, depending of distribution. In case significant difference is observed, post-hoc analysis is performed and *p* values are reported

## Discussion

In this study we describe specific resistance loops measured by body-plethysmography by using geometrical analyses and second-order transfer functions. The newly developed parameters reflect the curvilinear aspect and rotation of the resistance loops and typically associate with different lung function characteristics. Loops that are mathematically identified by an open appearance as measured by roundness typically occur in the majority of COPD subjects with hyperinflation, whereas rotation of the slope without opening is apparent during bronchoconstriction, as demonstrated in asthma.

Opening of the loops in COPD was significantly associated with RV/TLC ratio, much more than with FEV_1_ or FEV_1_/FVC ratio. In numerous studies the RV/TLC ratio has been linked to air trapping and hyperinflation, together with increases of RV and FRC [27–29]. In fact, the RV/TLC ratio reflects the proportion of trapped lung volume that cannot be mobilized by maximal breathing. Increases in static RV/TLC ratio are inversely correlated with maximal inspiratory capacity, which often declines during exercise by dynamic hyperinflation and strongly associates with breathing discomfort and dyspnea. From a mechanistic point of view, we can only speculate on the reasons why RV/TLC ratio is the best predictor for loop opening and asynchrony between alveolar pressures and flows. One possible explanation may be found in the competition for space between lung areas that are emptied during expiration with areas that are trapped with air and progressively compress adjacent airways during expiration. As such the heterogeneity of airflow may contribute to a wide distribution of time constants for gas emptying and thus asynchrony, typically occurring with hyperinflation. Heterogeneity in the ventilation may also occur following bronchoconstriction, but with limited hyperinflation. As methacholine challenge in the asthma group did not induce important asynchrony, one may hypothesize that a more homogenous reduction in flow was obtained. A last explanation may be found in increased airway collapse by reduced airway tethering with the loss of alveolar tissue. We did not observe any significant relationship between roundness and DL_CO_ or K_CO_, as indicative lung function markers of emphysema. Unfortunately, in the absence of CT measures we were not able to study the relationship with emphysema in more depth. From a clinical point of view the strong association between the roundness of resistance loops and hyperinflation is very attractive. The reduction of static hyperinflation is a main target of several COPD treatments including lung volume reduction surgery, endoscopic valve displacement and bronchodilators. Bronchodilator responses are usually evaluated on the expiratory volumes of spirometry, although different studies suggest that their impact on static lung volumes may be much larger [30–32]. Therefore, the monitoring of hyperinflation during tidal breathing may become a promising evaluation tool for treatment responses in specific phenotypes.

Our data clearly indicate that bronchoconstriction due to airway muscle contraction and mucosal oedema in asthma is resulting in significant changes in input related parameters such as SSG, point X, *b*_*0*_ and *b*_*1*_. These changes correspond to an increased volume shift in phase with the flow, which obviously result in a reduction of the slope. Although most of our asthma patients did not show opening, 4 subjects had significant increases in roundness after the bronchial challenge, which is not surprising as hyperinflation can occur during bronchoconstriction and heterogeneity in airflow during exhalation may occur with increased bronchoconstriction [33]. When using sG_AW_ as best correlate for the slope, we accept that volume shift and airflow have a linear relationship, which is often not the case. *SSG*, which is highly correlated to sG_AW_, accounts for pressure multiplication to reach the level of exhaled flow but under steady state condition. Both SSG and sG_AW_ ignore much of the system’s dynamics, but as they are computed differently they may still be influenced by other factors in the flow volume shift relationship. Looking at the slope *per se*, our data suggest to use sG_0.5_ instead of or with sG_AW_ (computed as sG_mid_) due to its potential to discriminate obstructive asthma from mild COPD. As sG_0.5_ practically represents the slope of the right hand side of the loop, it will be little influenced by resistive mechanisms that open the loop.

Previous studies with FOT have demonstrated that resistance measures can vary within and between breath as they are flow- and volume dependent. In particular, bronchomotor challenge can alter the ventilation which translates into changes of airway resistance and obscures the true changes in airway patency. Moreover, it has been shown that in patients with COPD the degree of flow limitation is varying between breaths [12, 34]. In the transition period of mild disease the detection of flow limitation may therefore require the monitoring of several breathing loops. For FOT different mathematical models have been proposed that correct for loop and flow dependency during tidal breathing [12, 15]. Our approach with plethysmography is adjusting flows for pressure changes during the whole breathing cycle and is taking the non-linearity of these dynamics into account. Indirectly, it also provides correction for between-breath differences as a representative breathing cycle of overlapping loops was carefully selected. Taken together, our data imply that airways are not just simple tubes, but flexible structures that may enlarge or compress in function of the manoeuver.

The new parameters that we have developed are potentially important as they are able to identify different phenotypes within the spectrum of obstructive airways diseases. Hence, it is only with interventions and longitudinal follow-up that we will be able to identify their exact clinical relevance. One weakness of the study is the smaller number of healthy subjects, since a larger number would secure confidence in defining the normality range for each parameter. Similarly, comparisons of postbronchodilator with prebronchodilator measures, particularly in asthma may reveal more subtle differences between asthma and healthy controls. Another weakness may be the lack of data from CT scans, as that would allow the correlation of newly developed parameters with CT parameters of hyperinflation, airtrapping, bronchial inflammation and emphysema [35–37].

## Conclusion

We developed new parameters which are reflecting the dynamic relationships between alveolar pressures and flows. Some of them are potentially important as they are linked to specific phenotypes within the spectrum of obstructive airways diseases. Mechanistic and prospective follow-up studies are now needed to determine the true validity of these parameters in respiratory medicine.
